# Negative linear compressibility in a crystal of α-BiB_3_O_6_

**DOI:** 10.1038/srep13432

**Published:** 2015-08-25

**Authors:** Lei Kang, Xingxing Jiang, Siyang Luo, Pifu Gong, Wei Li, Xiang Wu, Yanchun Li, Xiaodong Li, Chuangtian Chen, Zheshuai Lin

**Affiliations:** 1 Beijing Center for Crystal R&D, Key Lab of Functional Crystals and Laser Technology of Chinese Academy of Sciences, Technical Institute of Physics and Chemistry, Chinese Academy of Sciences, Beijing 100190, PR China; 2 School of Physics and Wuhan National High Magnetic Field Center, Huazhong University of Science and Technology, Wuhan 430074, PR China; 3 School of Earth and Space Sciences, Peking University, Beijing, 100871, PR China; 4 Beijing Synchrotron Radiation Facility, Institute of High Energy Physics Chinese Academy of Science, Beijing, 100049, PR China; 5 University of Chinese Academy of Sciences, Beijing 100190, PR China

## Abstract

Negative linear compressibility (NLC), a rare and important mechanical effect with many application potentials, in a crystal of *α*-BiB_3_O_6_ (BIBO) is comprehensively investigated using first-principles calculations and high-pressure synchrotron X-ray diffraction experiments. The results indicate that the BIBO crystal exhibits the second largest NLC among all known inorganic materials over a broad pressure range. This unusual NLC behaviour is due to the rotation and displacement of the rigid [BO_3_] and [BO_4_] building units that result in hinge motion in an umbrella-like topology. More importantly, the parallel-polar lone-pair electrons on the Bi^3+^ cations act as “umbrella stands” to withstand the B-O hinges, thus significantly enhancing the NLC effect. BIBO presents a unique example of a “collapsible umbrella” mechanism for achieving NLC, which could be applied to other framework materials with lone-pair electrons.

Over the past three decades, borate systems have been extensively studied because of their important applications in laser science and technology^1^. The vast structural diversity in borates provides numerous opportunities to search for and design optical materials with desired functionalities^2^. In particular, the anisotropic frameworks formed by [BO_3_]/[BO_4_] building units can generate strong nonlinear optical (NLO) effects^3^, and some NLO borates with superior performance have been discovered^4^. Note that the structural anisotropy may also result in strong anisotropy in the mechanical properties. It is very likely that structural features in accordance with the famous “wine-rack” motif^5^ would be discovered in borates, which could give rise to abnormal expansion of one direction under hydrostatic pressure due to the strong framework anisotropy, thus resulting in a very unusual negative linear compressibility (NLC) behaviour.

NLC is of great scientific interest because it contradicts the common knowledge that a material should shrink in all directions in response to hydrostatic pressure. The incompressibility along the NLC direction in these materials has many potential applications under high-pressure conditions, e.g., optical telecommunication devices that must function at deep-sea pressures, highly sensitive pressure detectors, force amplifiers, and shock-absorbing materials^5^,^6^,^7^,^8^. Regarding practicality, the mechanical robustness of inorganic materials has many advantages over organic and hybrid NLC materials. Hintherto, a handful of inorganic NLC systems have been discovered, including elemental Se^9^, LaNbO_4_^10^, and a few cyanides (e.g., Ag_3_[Co(CN)_6_]^11^ and KMn[Ag(CN)_2_]_3_^12^). In the borate system, the NLC effect has been extensively studied in BPO_4_ and BAsO_4_ only in terms of both phenomenon and underlying mechanism^13^.

In order to push the development of inorganic NLC materials forward^6^,^7^,^8^,^9^,^10^,^11^,^12^,^13^,^14^,^15^,^16^,^17^, the exploration of more NLC borates is highly sought after. Therefore, we screen all framework borates in the Inorganic Crystal Structure Database (ICSD, 2014-1, Version 1.9.4, by Fachinformatiionszentrum Karlsruhe, Germany) and conduct structural analyses. It is revealed that the monoclinic bismuth triborate BiB_3_O_6_ (*α*-BiB_3_O_6_, or BIBO)^18^ possesses significant framework anisotropy (see Fig. 1) which results in the anisotropic thermal, piezoelectric and mechanical properties^19^,^20^,^21^. In particular, BIBO has been previously observed to show NLC effect along its *a*-axis by Haussuhl *et al*.^20^ and Dinnebier *et al*.^21^. Their experiments revealed that the NLC behaviour is extraordinarily large (~ –12.5 TPa^−1^). It seems that the “wine-rack” mechanism alone is unlikely to produce such large NLC effect; the detailed investigation on the origin of NLC in BIBO is highly necessary. In this work, we perform first-principles calculations on the high-pressure behaviour of BIBO, and we highlight the strong NLC effect along its *a*-axis from ambient pressure to 6.5 GPa. This novel mechanical behaviour is subsequently confirmed through high-pressure X-ray diffraction (XRD) experiments. Moreover, the underlying mechanism for NLC in BIBO is elucidated and proposed as a so-called “collapsible umbrella” model, which would provide useful guidance for pursuing other NLC materials.

## Results

First, the full elastic tensors of BIBO (at 0 GPa) are determined using the finite strain technique^22^ based on first-principles calculations. The calculated elastic constants *C*_*ij*_ and the available experimental values from resonant ultrasound spectroscopy (RUS)^20^ are listed in Table 1 . To provide a better comparison, the experimental values that were measured at 293 K are extrapolated to 0 K using the formula *C*_*ij*_(0K) = *C*_*ij*_(*T*)exp(−*T*_*ij*_*·T*), where *T*_*ij*_ is the experimental thermoelastic constant and *T* = 293 K. Clearly, a good agreement between the experimental and calculated *C*_*ij*_ at 0 K is obtained. Furthermore, the linear compressibility *β*_*l*_ along the principal axes (*l* = *x*, *y*, and *z*) and the volume compressibility *β*_*V*_ transformed by the experimental *C*_*ij*_ (0 K) are in good agreement with the calculated values (see Table I). All of these results demonstrate the validity of the first-principles studies on the mechanical properties in the BIBO structure and confirm that our computational methods are sufficiently accurate for the purposes of this study.

Furthermore, the cell parameters and atomic positions in the unit cell of BIBO under hydrostatic pressures varying from 0 to 10 GPa (in intervals of 0.2 GPa) were fully optimised using first-principles calculations. The calculated variations in the relative cell constants with respect to the hydrostatic pressure changes from 0 to 10 GPa are shown in Fig. 2(a) (the crystallographic data are listed in Tables S1 and S2 of the Supplementary Information). As shown in this figure, as the pressure is applied until approximately 6.5 GPa, the cell constant *a* in BIBO is increased, whereas the cell constants *b*, *c* and volume *V* are decreased, manifesting as typical NLC behaviour. After the critical pressure of 6.5 GPa, a structural phase transition occurs, *i.e*., the α phase changes to a new phase (namely, the ε phase) of BIBO, and then all cell parameters have positive compressibility. This first-order phase transition could also be theoretically characterised by the non-smooth discontinuities of total energy, enthalpy and optical band gap with a sudden change in the total volume (see Figure S1 of the Supplementary Information). The simulated data show very good agreement with our high-pressure XRD experiments, particularly along the *a*-axis (see Fig. 2(a) and Tables S1 and S2 of the Supplementary Information). Our results are in very good agreement with the previous measurements^21^ (see Fig. 2).

To more clearly display the quantities, the compressibilities along the principal axes^23^ of BIBO with respect to the applied pressures are listed in Table S3 of the Supplementary Information and plotted in Fig. 2(b). As shown, the experimental (calculated) NLC *β*_*x*_ has a maximum of −23 TPa^−1^ (−27 TPa^−1^) at 0 GPa, and the value decreases as the pressure continuously increases with an average *β*_*x*_ of approximately −11.7 TPa^−1^ (−15.5 TPa^−1^) in the pressure interval of 0–2 GPa and of −6.4 TPa^–1^ (−6.9 TPa^−1^) over the entire NLC range of 0–6.5 GPa (see Figure S2 of the Supplementary Information). Regarding the very large NLC effect in BIBO, to the best of our knowledge, its NLC coefficient is just smaller than that of Zn[Au(CN)_2_]_2_ (−42 TPa^−1^ in the range of 0–1.8 GPa)^17^, which possesses the largest NLC coefficient among all known inorganic compounds in which NLC phenomena have been discovered, and considerably larger than that in the only other known borates BPO_4_ and BAsO_4_ (approximately −2 TPa^−1^ and −3 TPa^−1^, respectively, in the range of 5–40 GPa)^13^.

## Discussion

**Figure 1:**
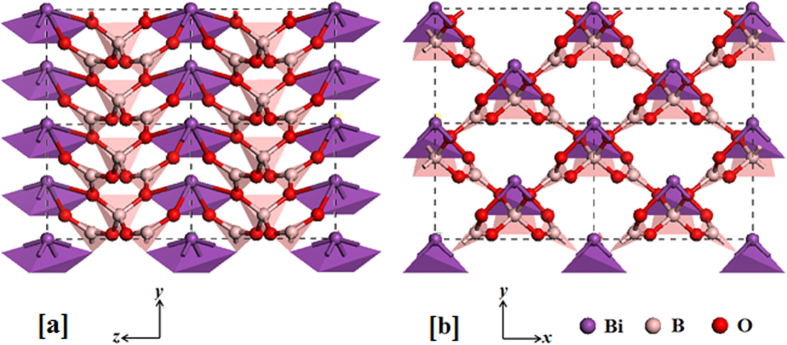
The framework structure of BIBO in the *y-z* plane(**a**) and the *x-y* plane (**b**) with ball-stick and polyhedron models (here, the crystallographic *c*-axis is along the *z*-axis, and the *b*-axis is in the *x*-*y* plane). Note that each [BO_4_] group is connected by corner sharing with two [BO_3_] groups in the *x*-*y* plane, and each bismuth ion is four-fold coordinated with the neighbouring oxygen atoms to form a [BiO_4_] pyramid. It was characterised in space group *C*2 with *a* = 7.120 Å, *b* = 4.995 Å, *c* = 6.508 Å, *α* = *γ* = 90°, and *β* = 105.59° (Ref. [18]).

**Figure 2:**
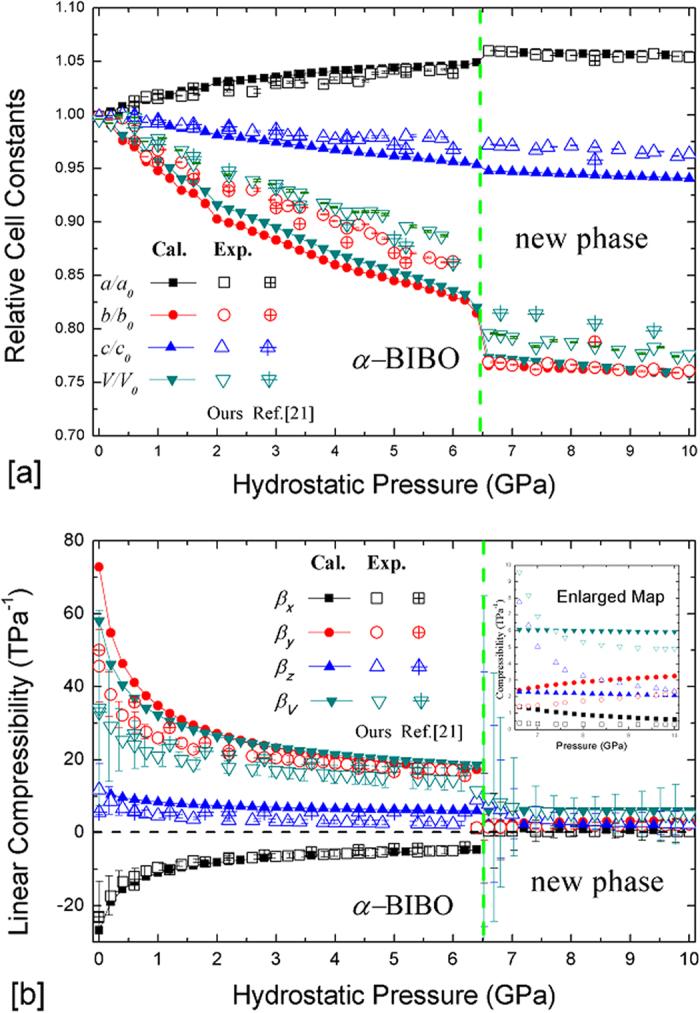
The experimental and calculated results of(**a**) relative cell constants *a/a*_*0*_, *b/b*_*0*_, *c/c*_*0*_ and *V/V*_*0*_ (*a*_*0*_, *b*_*0*_, *c*_*0*_ and *V*_*0*_ are the primitive values at 0 GPa) and (**b**) compressibility *β*_*x*_, *β*_*y*_, *β*_*z*_and *β*_*V*_ as a function of pressure from 0 to 10 GPa. The inset displays the enlarged linear compressibility of the new phase after 6.5 GPa. The experimental data from Ref. [21] are also included for comparison.

**Figure 3:**
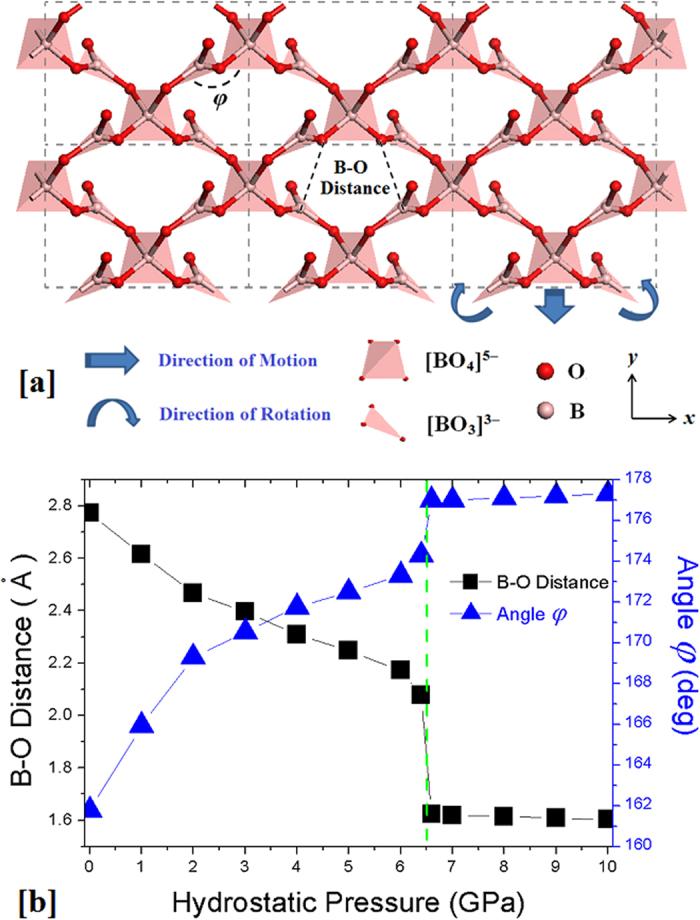
The borate framework of BIBO within the *x-y* plane(**a**) and the variation in the angle *φ* and B-O distance with respect to hydrostatic pressures of 0–10 GPa (**b**).

With the purpose of understanding the structural origins of NLC, the atomic geometries of BIBO under various hydrostatic pressures are theoretically investigated in detail. Compared with the structure at 0 GPa (Fig. 1), it is found that the bond lengths and angles within all [BO_3_] and [BO_4_] building units are almost constant (typically less than ± 2%) within the pressure interval of 0–6.5 GPa, *i.e*., the [BO_3_]/[BO_4_] groups appear to be “hard”, whereas the [BiO_4_] groups are relatively “soft” (see Figure S3 of the Supplementary Information). Thus, the borate framework structure, excluding the [BiO_4_] groups as shown in Fig. 3(a), is considered to be primarily responsible for the NLC abnormalities. Specifically, the angle (*φ*) between the [BO_3_]^3–^ triangles and [BO_4_]^5–^ tetrahedra within the *x-y* plane (marked in Fig. 3(a)) is responsible for the abnormal expansion along the *x*-axis when pressure is applied. As shown in Fig. 3(b), *φ* is initially approximately 161.8° at 0 GPa, and then it gradually increases until 6.4 GPa (~174.3°). This makes the [BO_3_-BO_4_-BO_3_] stripes flatten along the *x*-axis, and the entire lattice is expanded along the same direction (the structures under four different pressures are shown in Figure S4 of the Supplementary Information). Indeed, this NLC behaviour becomes smaller and terminates as the angle *φ* approaches approximately 180° (177.1° at 6.6 GPa, see Fig. 3(b)). At the same time, the boron atoms in the [BO_3_] triangle move outward from the plane as the pressure increases and gradually approach the oxygen atom in the nearby [BO_3_] triangle along the *y*-axis (see the B-O distance labelled in Fig. 3(a) and its variation shown in Fig. 3(b)). When the hydrostatic pressure is across the phase transition point (~6.5 GPa), the distances between these B and O atoms sharply decrease (from ~2.1 Å to ~1.6 Å), and the planar-like [BO_3_] groups transform into new tetrahedral [BO_4_] groups. This denser atomic structure of the new phase with parallel arranged [BO_4_] tetrahedra maintains the larger pressures until the end of our simulations at 10 GPa; thus, the NLC is frustrated in this phase.

The results of the above analysis reveal that the structural modifications of the angle *φ* mainly determine the NLC behaviour in BIBO. In the open framework, the periodic [BO_3_-BO_4_-BO_3_] unit is simply constructed by one [BO_4_]^5−^ tetrahedron on the top and two [BO_3_]^3−^ triangles on the bottom through sharing of the corner oxygen atoms, analogous to the shape of an “umbrella”. When this structure is compressed under an applied hydrostatic pressure, because the initial angle *φ* between the [BO_4_] tripod and [BO_3_] triangle is 162° (larger than 135°), the resulting forces on the bottom are not parallel to the edge of the triangle and its two “[BO_3_] leaves” are thus “opened up” due to the rotational freedom produced by the torque (or hinge motion, see the arrows of rotation in Fig. 3(a)). Thus, the crystal lattice typically contracts in the *y*-axis direction but expands in the perpendicular *x*-axis direction (see the arrows of motion). In a series of quasi-static procedures, the increase of *φ* results in the continuous expansion of the rigid “[BO_3_] leaves” projected on the *x*-axis until *φ* is close to 180°. However, if the angle *φ* was initially smaller than 135°, the direction of rotation for the “leaves” would be reversed such that the expansion would occur along the *y*-axis. Therefore, the origin of this NLC behaviour can be intuitively represented as a unique figurative “collapsible umbrella” mechanism in which the [BO_4_] “tripods” and rotatable [BO_3_] “leaves” result in a special open-framework topology for the generation of a NLC response in BIBO. Note that the NLC mechanism in BIBO is very different from that in BPO_4_ and BAsO_4_, where the corner-sharing tetrahedra are tilted to make the crystal gradually collapse from the open cristobalite-like framework to a dense structure^13^.

In addition, note that although the [BiO_4_] pyramids are not primarily involved in the NLC of BIBO, they are very important for the existence of this abnormal mechanical property in this special system. The stereochemical lone-pair electrons on the Bi^3+^ cations, which can be obtained by the electron localisation function (ELF) analysis^24^, not only stabilise the structural networks by filling in the interstices but also result in a relatively open framework such that the B-O groups have sufficient space to expand. In other words, the parallel-polar lone-pair electrons act as the “umbrella stands” to enhance the rigidity in the B-O groups (“umbrella surfaces”) and enlarge their interspace that gives rise to the relatively larger NLC effect, both in magnitude and in pressure range. The involvement of this electronic contribution is the main difference of the “collapsible umbrella” mechanism from the commonly recognized “wine-rack”^5^ or “Nuremberg scissors”^21^ mechanism in which only the atomic framework geometry is concerned. In order to better understand the difference, we construct a hypothetical crystal LaB_3_O_6_, where the Bi^3+^ cations (with the lone-pair electrons) in the BIBO structure are replaced by the La^3+^ cations (with the similar ionic radii but without the lone-pair electrons), so that the “wine-rack” mechanism is considered only. Our simulations demonstrate that the pure “wine-rack” motif in LaB_3_O_6_ results in a much smaller NLC *β*_*x*_ of ~ −2 TPa^−1^ and a much narrower NLC pressure range of ~1.5 GPa compared with the *β*_*x*_ value of −27 TPa^−1^ and the pressure range of 6.5 GPa in the “collapsible umbrella” structure in BIBO (see Table S4 and Figure S5).

In addition, note that due to technical limitations in the high-pressure measurements, the XRD patterns could only be obtained over a small 2*θ* range (less than 30°). Because of this limitation, the detailed atomic structures, particularly information regarding the new phase after ~6.5 GPa, cannot be directly determined from the XRD experiments. In a previous experiment^21^, it was concluded that the structural phase transition in BIBO that occurs between 6.09–6.86 GPa is attributed to the reorientation of the [BO_3_] triangles, the [BO_4_] tetrahedra and the lone-pair electrons localised at Bi^3+^ cations (*i.e*., a displacive phase transition). However, our simulations reveal that the formation of new [BO_4_] tetrahedra from the [BO_3_] triangles induces the phase transition (*i.e*., a reconstructed phase transition), while the orientation of the lone-pair electrons remains unchanged before and after the occurrence of the phase transition (see Figure S4 of the Supplementary Information). We also simulated the XRD pattern of our newly obtained structure, and we found that it also matches well with the experimental data; it is difficult to draw an absolute conclusion at this stagewhich phase, the previously simulated^21^ or ours, is in better agreement (see Figure S6 of the Supplementary Information). Interestingly, the total energy of our predicted structure is approximately 100 meV/atom lower than that of the structure in Ref. [21], which suggests that the former would be more stable under hydrostatic pressures of >6.5 GPa. We suggest that the crystal structure of BIBO after the phase transition should be re-determined if the experimental conditions were available.

In conclusion, we focused on the NLC effect in the borate system because of its extraordinary structural diversity and versatility. Considering that thousands of borate compounds have been discovered, it is considerably more efficient to identify one type of borate as a promising NLC material and more effective to understand the intrinsic mechanism via atomic simulations. As a good beginning, the comprehensive first-principles calculations on the mechanical properties of BIBO are in very good agreement with the experimental results obtained from high-pressure XRD and RUS measurements, which confirm that BIBO exhibits a large and persistent NLC response. More importantly, in our present work, the mechanism of the anomalous mechanical effect is elucidated in detail. Namely, the singular NLC of BIBO arises from the hinge motion of the rigid [BO_3_] and [BO_4_] building units as well as the singnificant contribution of the parallel-polar lone-pair electrons on the Bi^3+^ cations in the “collapsible umbrella” structure. This NLC mechanism with the synergistic effect of framework topology and lone-pair electrons is discovered for the first time and would effectively enhance the efficiency in searching for NLC materials. Finally, as an applied inorganic nonlinear optical material, a large and high quality BIBO crystal has been grown with good chemical stability and mechanical properties^25^, which is very important for the potential applications of any functional material. All of these analyses associate and link with each other to build and constitute a complete study on the NLC characteristics of the BIBO crystal, which will not only greatly extend the understanding of the NLC response but also promote interest in explorations on NLC in the framework materials with lone-pair electrons.

## Methods

The first-principles calculations were performed using the CASTEP package^26^ with the ultrasoft pseudopotentials^27^ and the local density approximation^28^, which has been employed to accurately study the physiochemical properties of borates^29^. A kinetic energy cut-off of 500 eV and Monkhorst-Pack *k*-point meshes spanning less than 0.04/Å in the Brillouin zone were selected for all of the calculations. For the experimental measurements, high-pressure XRD patterns of very fine powder samples were recorded at the Beijing Synchrotron Radiation Facility. Synchrotron X-rays with a wavelength of 0.61992 Å was focused in the horizontal and vertical directions onto a 36  ×  12  μm^2^ spot using Kirkpatrick–Baez mirrors. The hydrostatic pressure on the BIBO samples was exerted by a diamond anvil cell. The measured pressure range was from 0 to 12 GPa in intervals of approximately 0.3 GPa. The cell parameters under different pressures were determined from Rietveld refinement^30^ as implemented in the GSAS software package^31^.

## Additional Information

**How to cite this article**: Kang, L. *et al*. Negative linear compressibility in a crystal of α-BiB_3_O_6_. *Sci. Rep*. **5**, 13432; doi: 10.1038/srep13432 (2015).

## Supplementary Material

Supplementary Information

## Figures and Tables

**Table 1:** Comparison between experimental and calculated elastic constants *C*_*ij*_ (GPa) and compressibility coefficients *β*_*x*_*, β*_*y*_*, β*_*z*_, and *β*_*V*_ (GPa^−1^) of BIBO.

Experimental values at 293 K and 0 K				Calculated
*C*_*ij*_ (293 K)^a^		*T*_*ij*_ (10^−3^ K^−1^)^a^	*C*_*ij*_ (0 K)	*C*_*ij*_ (0 K)
*C*_*11*_	159.70 (60)	–0.22 (5)	170.33	183.23
*C*_*22*_	52.50 (60)	–0.25 (5)	56.49	49.11
*C*_*33*_	205.20 (70)	–0.17 (4)	215.68	239.33
*C*_*44*_	23.30 (30)	–0.16 (4)	24.42	18.13
*C*_*55*_	74.60 (40)	–0.23 (4)	79.80	99.29
*C*_*66*_	66.90 (40)	–0.12 (3)	69.29	65.32
*C*_*12*_	74.20 (100)	–0.21 (5)	78.91	66.64
*C*_*13*_	60.00 (80)	–0.10 (1)	61.78	65.44
*C*_*23*_	13.40 (200)	–0.40 (7)	15.07	5.34
*C*_*15*_	–49.70 (60)	–0.13 (9)	–51.63	–55.21
*C*_*25*_	–4.30 (120)	–0.10 (7)	–4.43	4.67
*C*_*35*_	–70.80 (60)	–0.19 (5)	–74.85	–81.68
*C*_*46*_	–18.60 (40)	–0.06 (6)	–18.93	–23.98
Compressibility coefficients *β*_*x*_, *β*_*y*_, *β*_*z*_, and *β*_*V*_				
Exp. at 0 K (GPa^–1^)		Cal. at 0 K (GPa^–1^)		
*β*_*x *_= –10.10 × 10^–3^		*β*_*x*_ = –8.30 × 10^–3^		
*β*_*y*_ = 30.40 × 10^–3^		*β*_*y*_ = 31.24 × 10^–3^		
*β*_*z*_ = 5.60 × 10^–3^		*β*_*z*_ = 5.11 × 10^–3^		
*β*_*V *_= 25.90 × 10^–3^		*β*_*V*_ = 28.05 × 10^–3^		

Here, a transformation from the crystallographic coordination to the principal coordination (*a, b, c* → *x*, *y* and *z*) is performed.

^a^Reference [20].
