# Supplementary material for: Negative linear compressibility in a crystal of α-BiB3O6
Source: Sci Rep. 2015 Aug 25;5:13432. doi: 10.1038/srep13432 (PMC4548252; doi:10.1038/srep13432)
Supplement: Supplementary Information [file srep13432-s1.pdf]

Supplementary Information for

Negative linear compressibility in a crystal of α-BiB_3O_6

Lei Kang, [€,†,¶] **Xingxing Jiang,**[€,†,¶] **Siyang Luo,**[†] **Pifu Gong,**[†,¶] **Wei Li,**[*,‡] **Xiang Wu,**[§] **Yanchun Li,**[‖] **Xiaodong Li,**[‖] **Chuangtian Chen**[†], **and Zheshuai Lin**[*,†]

[†] Beijing Center for Crystal R&D, Key Lab of Functional Crystals and Laser Technology of Chinese Academy of Sciences, Technical Institute of Physics and Chemistry, Chinese Academy of Sciences, Beijing 100190, PR China

[‡] School of Physics and Wuhan National High Magnetic Field Center, Huazhong University of Science and Technology, Wuhan 430074, PR China

[§] School of Earth and Space Sciences, Peking University, Beijing, 100871, PR China

[‖] Institute of High Energy Physics Chinese Academy of Science, Beijing, 100049, PR China

[¶] University of Chinese Academy of Sciences, Beijing 100190, PR China

[€] L. K. and X. X. J. contribute equally to this work.

*Corresponding authors: wl276@hust.edu.cn (W. L.) and zslin@mail.ipc.ac.cn (Z. S. L.)

Content

1. **Table S1.** The computed crystallographic data of the optimized BIBO crystals under the hydrostatic pressure varying from 0 to 10 GPa.
2. **Table S2.** The crystallographic data of BIBO from high-pressure X-ray diffraction (XRD) experiments under different hydrostatic pressures from 0 to 12 GPa. The errors are given in the parentheses.
3. **Figure S1.** The calculated total energy [a], enthalpy [b], optical band gap [c], and β angle of cell parameter [d] as the function of pressure from 0 to 10 GPa, respectively.
4. **Table S3.** The experimental and calculated results of compressibility β_x, β_y, β_z and β_V as a function of pressure from 0 to 10 GPa. The errors are given in the parentheses.
5. **Figure S2.** The experimental compressibility indicatrix in the range of 0 – 2 GPa [a] and 0 – 6.5 GPa [b], and the calculated ones in the same range of 0 – 2 GPa [c] and 0 – 6.5 GPa [d].
6. **Figure S3.** The average relative bond lengths [a] and bond angles [b] of $[BO_3]^{3-}$/$[BO_4]^{5-}$/$[BiO_4]^{5-}$ groups under different hydrostatic pressures from 0 to 10 GPa.
7. **Figure S4.** The atomic structures of BIBO in the *x*-*y* plane under different pressures: [a] 3.0 GPa, [b] 6.4 GPa, [c] 6.6 GPa, and [d] 9.0 GPa.
8. **Table S4.** Calculated elastic constants C_{ij} (GPa) and compressibility coefficients β_x, β_y, β_z, and β_V (GPa^{-1}) of LaB_3O_6.
9. **Figure S5.** The calculated mechanical properties under pressure in the hypothetical crystal LaB_3O_6 which is geometrically optimized from α-BiB_3O_6 by replacing the Bi^{3+} cations with La^{3+} cations: [a] relative cell constants a/a_0, b/b_0, c/c_0 and V/V_0 (a_0, b_0, c_0 and V_0 are the primitive values at 0 GPa) and [b] compressibility β_x, β_y, β_z and β_V as a function of pressure from 0 to 10 GPa.
10. **Figure S6.** The comparison between the experimental XRD pattern and those simulated from the ε-BIBO in Ref. 4 [a] and in this work [b].
11. **References**

1. **Table S1.** The cell parameters and atomic position in unit cell of BIBO under the hydrostatic pressures varied from 0 to 10 GPa (with the interval of 0.2 GPa) are fully optimized using the quasi-Newton method[1]. The convergence thresholds between optimization cycles for energy change, maximum force, maximum stress, and maximum displacement are set as 5.0×10^{-6} eV per atom, 0.01 eV per Å, 0.02 GPa, and 5.0×10^{-4} Å, respectively. The optimization terminates when all of these criteria are satisfied. The computed crystallographic data of the optimized BIBO crystals under different hydrostatic pressure are listed as follows.

Pressure (GPa)	a (Å)	b (Å)	c (Å)	α (deg)	β (deg)	γ (deg)	V (Å^3)
0	7.100	4.808	6.338	90.00	103.97	90.00	209.995
0.2	7.121	4.763	6.331	90.00	103.85	90.00	208.488
0.4	7.155	4.694	6.316	89.99	103.75	90.00	206.022
0.6	7.167	4.665	6.309	90.00	103.68	90.00	204.924
0.8	7.200	4.598	6.293	90.00	103.57	90.00	202.505
1.0	7.217	4.558	6.284	90.01	103.49	89.99	200.999
1.2	7.231	4.524	6.274	90.00	103.44	90.00	199.646
1.4	7.257	4.469	6.258	90.00	103.36	90.00	197.491
1.6	7.260	4.456	6.254	90.00	103.33	90.00	196.901
1.8	7.281	4.410	6.240	90.00	103.24	90.00	195.076
2.0	7.319	4.340	6.219	90.00	103.10	90.00	192.398
2.2	7.325	4.323	6.210	90.00	103.11	90.00	191.507
2.4	7.328	4.310	6.204	90.00	103.08	90.00	190.886
2.6	7.338	4.285	6.196	90.00	103.02	90.00	189.811
2.8	7.345	4.268	6.185	90.00	103.02	90.00	188.913
3.0	7.353	4.246	6.176	90.00	102.98	90.00	187.894
3.2	7.361	4.225	6.168	90.00	102.94	90.00	186.940
3.4	7.371	4.200	6.157	90.00	102.90	90.00	185.796
3.6	7.377	4.181	6.150	90.00	102.86	90.00	184.907
3.8	7.385	4.157	6.140	90.00	102.81	90.00	183.785
4.0	7.392	4.135	6.130	90.00	102.78	90.00	182.756
4.2	7.396	4.122	6.124	90.00	102.75	90.00	182.098
4.4	7.402	4.104	6.116	90.00	102.71	90.00	181.229
4.6	7.404	4.094	6.111	90.00	102.71	90.00	180.686
4.8	7.406	4.081	6.104	90.00	102.66	90.00	180.020
5.0	7.413	4.064	6.095	90.00	102.64	90.00	179.180
5.2	7.415	4.053	6.091	90.00	102.62	90.00	178.606
5.4	7.417	4.041	6.084	90.00	102.61	90.01	177.943
5.6	7.422	4.024	6.077	90.00	102.57	90.00	177.166
5.8	7.425	4.009	6.070	90.00	102.55	90.00	176.364
6.0	7.431	3.990	6.062	90.00	102.50	90.00	175.501
6.2	7.433	3.976	6.057	90.00	102.47	90.00	174.779
6.4	7.449	3.918	6.043	90.00	102.35	90.00	172.271
6.6	7.518	3.682	6.006	90.00	102.35	90.00	162.416
6.8	7.515	3.681	6.006	90.00	102.34	90.00	162.311
7.0	7.515	3.678	6.002	90.00	102.34	90.00	162.070
7.2	7.512	3.677	6.000	90.00	102.34	90.00	161.890
7.4	7.512	3.674	5.997	90.00	102.36	90.00	161.694

7.6	7.510	3.672	5.994	90.00	102.35	90.00	161.471
7.8	7.508	3.670	5.991	90.00	102.35	90.00	161.267
8.0	7.507	3.668	5.988	90.00	102.35	90.00	161.074
8.2	7.506	3.666	5.986	90.00	102.35	90.00	160.880
8.4	7.504	3.664	5.983	90.00	102.36	90.00	160.673
8.6	7.503	3.662	5.980	90.00	102.36	90.00	160.491
8.8	7.502	3.660	5.977	90.00	102.36	90.00	160.294
9.0	7.500	3.658	5.975	90.00	102.37	90.00	160.103
9.2	7.501	3.654	5.973	90.00	102.35	90.00	159.907
9.4	7.499	3.652	5.971	90.00	102.34	90.00	159.741
9.6	7.498	3.650	5.968	90.00	102.35	90.00	159.552
9.8	7.496	3.648	5.966	90.00	102.35	90.00	159.388
10.0	7.495	3.646	5.963	90.00	102.35	90.00	159.184

2. **Table S2.** The crystallographic data of BIBO from high-pressure X-ray diffraction (XRD) experiments under different hydrostatic pressures from 0 to 12 GPa. The errors are given in the parentheses.

Pressure (GPa)	a (Å)	b (Å)	c (Å)	α (deg)	β (deg)	γ (deg)	V (Å^3)
0.00	7.1049 (5)	4.9865 (7)	6.4968 (9)	90.00	105.524 (10)	90.00	221.776 (33)
0.17	7.1104 (10)	4.9695 (20)	6.4904 (22)	90.00	105.579 (21)	90.00	220.91 (8)
0.33	7.1274 (16)	4.9768 (25)	6.5105 (24)	90.00	105.562 (35)	90.00	222.48 (13)
0.60	7.1645 (7)	4.9423 (16)	6.5070 (9)	90.00	105.419 (20)	90.00	222.11 (5)
0.77	7.2372 (6)	4.7976 (13)	6.4602 (12)	90.00	104.562 (35)	90.00	217.10 (5)
1.07	7.2261 (25)	4.8284 (26)	6.4611 (15)	90.00	104.973 (33)	90.00	217.78 (10)
1.35	7.2425 (24)	4.7718 (23)	6.4452 (12)	90.00	104.835 (22)	90.00	215.32 (9)
1.65	7.2486 (34)	4.7182 (22)	6.4309 (14)	90.00	104.845 (31)	90.00	212.60 (9)
2.17	7.276 (9)	4.659 (5)	6.421 (4)	90.00	105.09 (9)	90.00	210.17 (18)
2.67	7.272 (13)	4.643 (8)	6.407 (4)	90.00	104.95 (11)	90.00	209.02 (28)
2.92	7.326 (5)	4.5948 (25)	6.3891 (14)	90.00	104.47 (4)	90.00	208.23 (9)
3.23	7.331 (5)	4.5686 (23)	6.3794 (13)	90.00	104.465 (5)	90.00	206.89 (33)
3.48	7.329 (4)	4.5607 (22)	6.3798 (12)	90.00	104.503 (32)	90.00	206.46 (8)
3.81	7.320 (5)	4.5274 (25)	6.3715 (13)	90.00	104.629 (34)	90.00	204.32 (9)
3.98	7.357 (8)	4.496 (4)	6.3582 (19)	90.00	104.68 (6)	90.00	203.45 (16)
4.34	7.360 (5)	4.4636 (33)	6.3740 (16)	90.00	104.83 (5)	90.00	202.42 (15)
4.51	7.340 (5)	4.4801 (30)	6.360 (5)	90.00	104.53 (6)	90.00	202.47 (17)
4.67	7.390 (6)	4.441 (4)	6.372 (6)	90.00	104.39 (7)	90.00	202.55 (21)
4.84	7.401 (8)	4.415 (5)	6.375 (7)	90.00	104.17 (8)	90.00	201.94 (26)
5.31	7.416 (8)	4.347 (4)	6.381 (7)	90.00	104.15 (8)	90.00	199.44 (26)
5.57	7.412 (6)	4.3266 (32)	6.372 (5)	90.00	103.83 (6)	90.00	198.42 (20)
5.85	7.417 (7)	4.3032 (31)	6.369 (6)	90.00	103.66 (7)	90.00	197.51 (20)
6.13	7.441 (8)	4.2788 (26)	6.395 (6)	90.00	103.38 (7)	90.00	198.10 (20)
6.38	7.5171 (12)	3.8375 (28)	6.3271 (24)	97.04 (8)	102.757 (29)	91.04 (6)	176.40 (14)
6.52	7.5183 (11)	3.8363 (28)	6.3229 (25)	97.19 (8)	102.778 (29)	91.04 (6)	176.25 (14)
6.69	7.5421 (11)	3.8392 (29)	6.3263 (25)	96.49 (10)	102.769 (30)	91.78 (6)	177.21 (14)
6.80	7.5403 (11)	3.8349 (29)	6.3218 (24)	96.45 (10)	102.766 (29)	91.81 (6)	176.86 (14)
7.02	7.5349 (14)	3.825 (4)	6.3204 (31)	96.03 (18)	102.76 (4)	91.90 (10)	176.36 (20)
7.39	7.5177 (13)	3.806 (4)	6.2952 (31)	95.89 (14)	102.79 (4)	91.93 (9)	174.45 (18)
7.70	7.5094 (15)	3.8339 (35)	6.3126 (34)	97.22 (10)	102.81 (4)	90.71 (8)	175.67 (18)
8.04	7.5104 (18)	3.8260 (23)	6.3207 (19)	97.45 (6)	102.917 (33)	90.83 (6)	175.36 (11)
8.40	7.5075 (15)	3.8145 (20)	6.3087 (18)	97.26 (6)	102.897 (29)	91.14 (5)	174.48 (10)
8.74	7.5011 (16)	3.8047 (24)	6.2992 (24)	96.99 (8)	102.840 (34)	91.33 (6)	173.74 (12)
9.19	7.5030 (12)	3.7970 (34)	6.2722 (26)	95.80 (12)	102.913 (33)	91.81 (7)	173.01 (16)
9.51	7.5243 (11)	3.8075 (31)	6.2836 (24)	95.75 (10)	102.915 (30)	91.93 (7)	174.29 (15)
9.76	7.5042 (11)	3.7891 (25)	6.2566 (23)	95.58 (7)	102.808 (28)	91.77 (5)	172.40 (13)
10.22	7.5002 (14)	3.7990 (26)	6.2713 (27)	97.04 (8)	102.719 (33)	90.93 (6)	172.82 (13)
10.73	7.5010(10)	3.8089(26)	6.2582(23)	97.17(8)	102.825(28)	90.69(5)	172.83(13)
11.18	7.4966(16)	3.7751(21)	6.2573(20)	96.97(6)	102.644(32)	91.09(6)	171.32(10)
11.66	7.5203(18)	3.7828(23)	6.2678(20)	97.01(7)	102.696(34)	91.22(6)	172.44(11)
12.12	7.5269(19)	3.7785(22)	6.2530(19)	97.00(7)	102.70(4)	91.09(6)	172.01(11)

3. **Figure S1.** The calculated total energy [a], enthalpy [b], optical band gap [c], and β angle of cell parameter [d] as the function of pressure from 0 to 10 GPa, respectively.

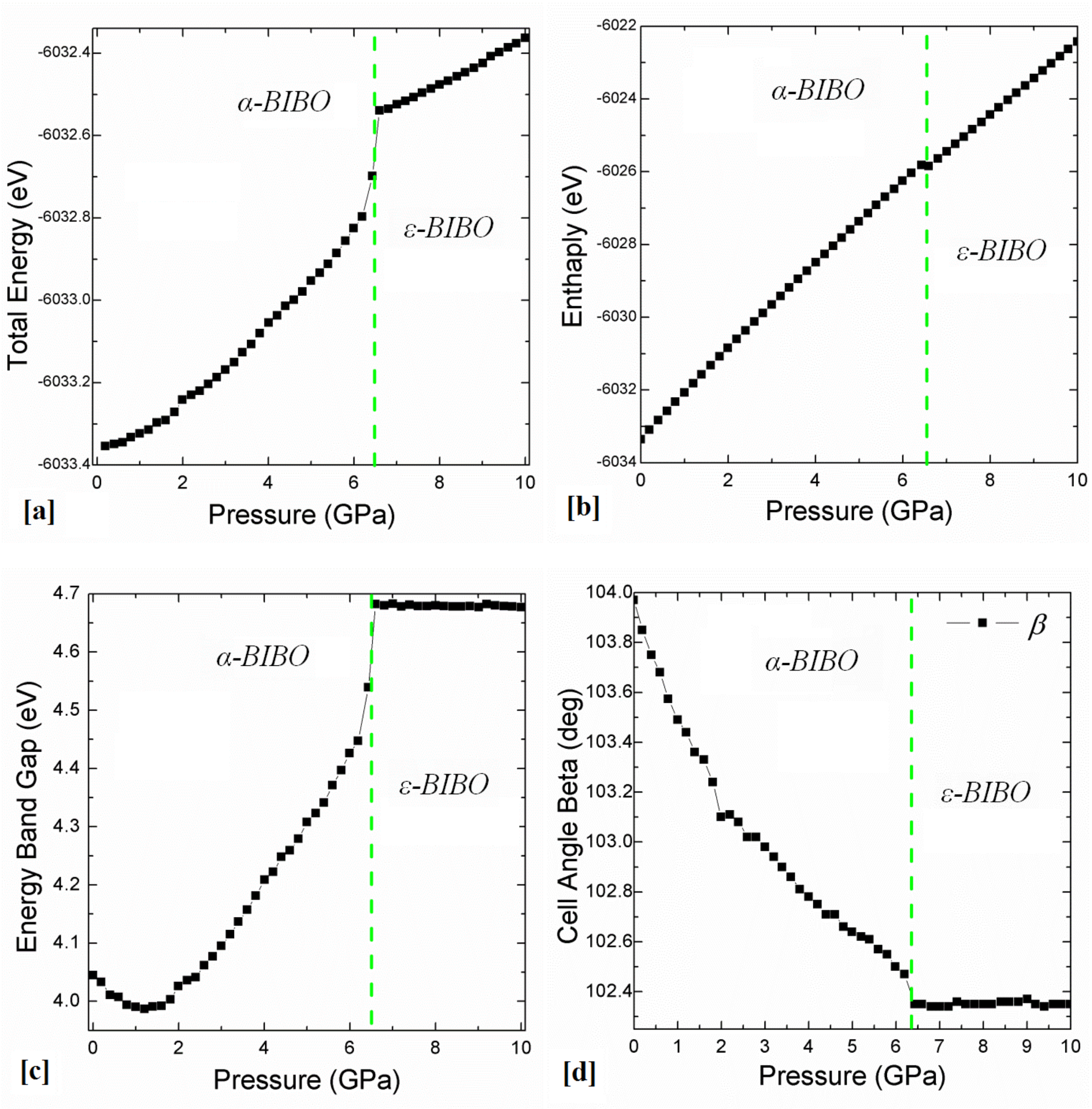

4. Table S3 The experimental and calculated results of compressibility β_x, β_y, β_z and β_V as a function of pressure from 0 to 10 GPa. The errors are given in the parentheses.

P (GPa)	Calculated results (TPa^{-1})				P (GPa)	Experimental results (TPa^{-1})			
	β_x	β_y	β_z	β_V		β_x	β_y	β_z	β_V
0.0	-26.76 (4.87)	72.81 (8.85)	11.99 (1.04)	58.04 (14.76)	0.00	-23.29 (9.86)	45.60 (9.93)	11.75 (6.98)	34.06 (26.77)
0.2	-18.99 (2.50)	54.73 (4.84)	10.45 (0.67)	46.19 (8.01)	0.17	-17.38 (5.41)	37.86 (6.12)	8.66 (3.77)	29.14 (15.30)
0.4	-15.53 (1.60)	46.31 (3.21)	9.64 (0.49)	40.42 (5.30)	0.33	-13.44 (2.88)	32.14 (3.64)	6.62 (1.98)	25.32 (8.50)
0.6	-13.47 (1.11)	41.14 (2.31)	9.10 (0.38)	36.77 (3.80)	0.60	-12.14 (2.14)	30.12 (2.84)	5.95 (1.47)	23.93 (6.45)
0.8	-12.06 (0.81)	37.53 (1.73)	8.70 (0.30)	34.17 (2.84)	0.77	-10.54 (1.34)	27.54 (1.90)	5.14 (0.91)	22.14 (4.18)
1.0	-11.02 (0.61)	34.81 (1.33)	8.39 (0.24)	32.18 (2.18)	1.07	-9.57 (0.92)	25.89 (1.37)	4.64 (0.63)	20.96 (2.92)
1.2	-10.21 (0.48)	32.67 (1.04)	8.14 (0.20)	30.60 (1.72)	--	--	--	--	--
1.4	-9.56 (0.38)	30.92 (0.83)	7.93 (0.16)	29.29 (1.37)	1.35	-8.78 (0.66)	24.52 (0.98)	4.24 (0.45)	19.98 (2.09)
1.6	-9.02 (0.31)	29.46 (0.68)	7.74 (0.13)	28.18 (1.12)	1.65	-7.81 (0.51)	22.76 (0.69)	3.76 (0.35)	18.71 (1.55)
1.8	-8.56 (0.27)	28.21 (0.58)	7.58 (0.11)	27.23 (0.96)	--	--	--	--	--
2.0	-8.16 (0.25)	27.12 (0.52)	7.44 (0.10)	26.40 (0.87)	--	--	--	--	--
2.2	-7.82 (0.24)	26.17 (0.50)	7.31 (0.09)	25.66 (0.83)	2.17	-7.15 (0.56)	21.52 (0.70)	3.43 (0.38)	17.80 (1.64)
2.4	-7.52 (0.24)	25.32 (0.49)	7.20 (0.08)	25.00 (0.81)	--	--	--	--	--
2.6	-7.25 (0.25)	24.56 (0.51)	7.09 (0.08)	24.40 (0.84)	2.67	-6.89 (0.60)	21.01 (0.76)	3.29 (0.41)	17.41 (1.77)
2.8	-7.00 (0.26)	23.87 (0.53)	6.99 (0.09)	23.86 (0.88)	--	--	--	--	--
3.0	-6.78 (0.27)	23.24 (0.56)	6.91 (0.09)	23.37 (0.92)	2.92	-6.60 (0.65)	20.45 (0.84)	3.15 (0.45)	17.00 (1.94)
3.2	-6.58 (0.28)	22.67 (0.59)	6.82 (0.10)	22.91 (0.97)	3.23	-6.40 (0.69)	20.05 (0.91)	3.05 (0.47)	16.70 (2.07)
3.4	-6.40 (0.30)	22.14 (0.61)	6.75 (0.10)	22.49 (1.01)	3.48	-6.15 (0.74)	19.56 (1.01)	2.93 (0.50)	16.34 (2.25)
3.6	-6.23 (0.31)	21.66 (0.64)	6.67 (0.11)	22.10 (1.06)	--	--	--	--	--
3.8	-6.07 (0.32)	21.20 (0.67)	6.61 (0.12)	21.74 (1.11)	3.81	-6.04 (0.76)	19.34 (1.05)	2.87 (0.52)	16.17 (2.33)
4.0	-5.93 (0.33)	20.78 (0.70)	6.54 (0.12)	21.39 (1.15)	3.98	-5.83 (0.81)	18.89 (1.14)	2.77 (0.55)	15.83 (2.50)
4.2	-5.79 (0.34)	20.39 (0.72)	6.48 (0.13)	21.08 (1.19)	4.34	-5.73 (0.83)	18.70 (1.18)	2.72 (0.56)	15.69 (2.57)
4.4	-5.67 (0.35)	20.02 (0.75)	6.42 (0.14)	20.77 (1.24)	4.51	-5.64 (0.84)	18.51 (1.22)	2.68 (0.57)	15.55 (2.63)
4.6	-5.55 (0.35)	19.67 (0.77)	6.37 (0.14)	20.49 (1.26)	4.67	-5.56 (0.86)	18.34 (1.25)	2.64 (0.58)	15.42 (2.69)
4.8	-5.44 (0.36)	19.34 (0.79)	6.32 (0.15)	20.22 (1.30)	4.84	-5.34 (0.90)	17.88 (1.35)	2.53 (0.61)	15.07 (2.86)
5.0	-5.33 (0.37)	19.03 (0.81)	6.27 (0.16)	19.97 (1.34)	--	--	--	--	--
5.2	-5.23 (0.37)	18.74 (0.83)	6.22 (0.16)	19.73 (1.36)	--	--	--	--	--
5.4	-5.14 (0.38)	18.46 (0.85)	6.18 (0.17)	19.50 (1.40)	5.31	-5.24 (0.92)	17.66 (1.39)	2.48 (0.62)	14.90 (2.93)
5.6	-5.05 (0.39)	18.20 (0.86)	6.13 (0.17)	19.28 (1.42)	5.57	-5.13 (0.94)	17.43 (1.44)	2.42 (0.63)	14.72 (3.01)
5.8	-4.97 (0.39)	17.94 (0.88)	6.09 (0.18)	19.06 (1.45)	5.85	-5.03 (0.96)	17.21 (1.48)	2.37 (0.65)	14.55 (3.09)
6.0	-4.89 (0.40)	17.70 (0.89)	6.05 (0.18)	18.86 (1.47)	6.13	-4.90 (0.98)	17.00 (1.52)	2.32 (0.67)	14.42 (3.17)
6.2	-4.81 (0.40)	17.47 (0.91)	6.02 (0.19)	18.68 (1.50)	6.38	--	--	--	--
6.4	-4.73 (0.41)	17.27 (0.92)	5.99 (0.26)	18.53 (1.52)	6.52	1.05 (15.00)	1.37 (3.50)	9.00 (34.89)	11.42 (54.39)
6.6	1.40 (0.39)	2.39 (0.57)	2.29 (0.18)	6.08 (1.22)	6.69	0.39 (10.27)	1.41 (2.25)	7.77 (21.47)	9.57 (33.99)
6.8	1.30 (0.22)	2.50 (0.29)	2.27 (0.13)	6.07 (0.69)	6.80	0.37 (7.95)	1.43 (1.92)	6.36 (13.42)	8.16 (23.29)
7.0	1.21 (0.12)	2.59 (0.15)	2.26 (0.08)	6.06 (0.40)	7.02	0.36 (5.26)	1.48 (1.33)	5.05 (6.88)	6.89 (13.47)
7.2	1.14 (0.07)	2.67 (0.09)	2.25 (0.06)	6.06 (0.24)	--	--	--	--	--
7.4	1.07 (0.06)	2.74 (0.08)	2.23 (0.05)	6.04 (0.20)	7.39	0.34 (2.93)	1.57 (0.71)	4.08 (2.99)	5.99 (6.63)
7.6	1.01 (0.06)	2.80 (0.08)	2.22 (0.06)	6.03 (0.19)	7.70	0.34 (1.86)	1.64 (0.65)	3.62 (1.86)	5.60 (4.37)
7.8	0.96 (0.07)	2.85 (0.08)	2.21 (0.06)	6.02 (0.21)	--	--	--	--	--
8.0	0.91 (0.06)	2.90 (0.08)	2.19 (0.06)	6.00 (0.20)	8.04	0.33 (1.38)	1.72 (0.82)	3.28 (1.77)	5.33 (3.97)

8.2	0.87 (0.05)	2.94 (0.07)	2.18 (0.06)	5.99 (0.18)	--	--	--	--	--
8.4	0.83 (0.04)	2.99 (0.06)	2.17 (0.06)	5.99 (0.16)	8.40	0.33 (1.57)	1.81 (0.92)	3.01 (2.12)	5.15 (6.18)
8.6	0.80 (0.04)	3.03 (0.05)	2.16 (0.05)	5.99 (0.14)	--	--	--	--	--
8.8	0.77 (0.04)	3.06 (0.05)	2.14 (0.05)	5.97 (0.14)	8.74	0.32 (1.99)	1.90 (0.84)	2.83 (2.47)	5.05 (5.30)
9.0	0.74 (0.05)	3.10 (0.06)	2.13 (0.05)	5.97 (0.16)	--	--	--	--	--
9.2	0.71 (0.06)	3.13 (0.08)	2.12 (0.06)	5.96 (0.20)	9.19	0.32 (2.58)	2.01 (0.72)	2.63 (2.86)	4.96 (6.16)
9.4	0.68 (0.08)	3.16 (0.10)	2.11 (0.08)	5.95 (0.18)	--	--	--	--	--
9.6	0.66 (0.09)	3.19 (0.12)	2.10 (0.12)	5.95 (0.26)	9.51	0.32 (2.96)	2.09 (1.06)	2.52 (3.08)	4.93 (7.10)
9.8	0.64 (0.11)	3.22 (0.15)	2.09 (0.16)	5.95 (0.33)	9.76	0.31 (3.25)	2.16 (1.67)	2.44 (3.23)	4.91 (8.15)
10.0	0.62 (0.13)	3.25 (0.18)	2.08 (0.20)	5.95 (0.42)	10.22	0.31 (3.71)	2.29 (3.27)	2.32 (3.45)	4.92 (10.43)

5. **Figure S2.** The experimental compressibility indicatrix in the range of 0 – 2 GPa [a] and 0 – 6.5 GPa [b], and the calculated ones in the same range of 0 – 2 GPa [c] and 0 – 6.5 GPa [d]. In the blank, the magnitudes of the corresponding average compressibility β_x, β_y and β_z are listed. The blue values correspond to negative linear compressibility (NLC) and the red ones to positive linear compressibility (PLC)[2].

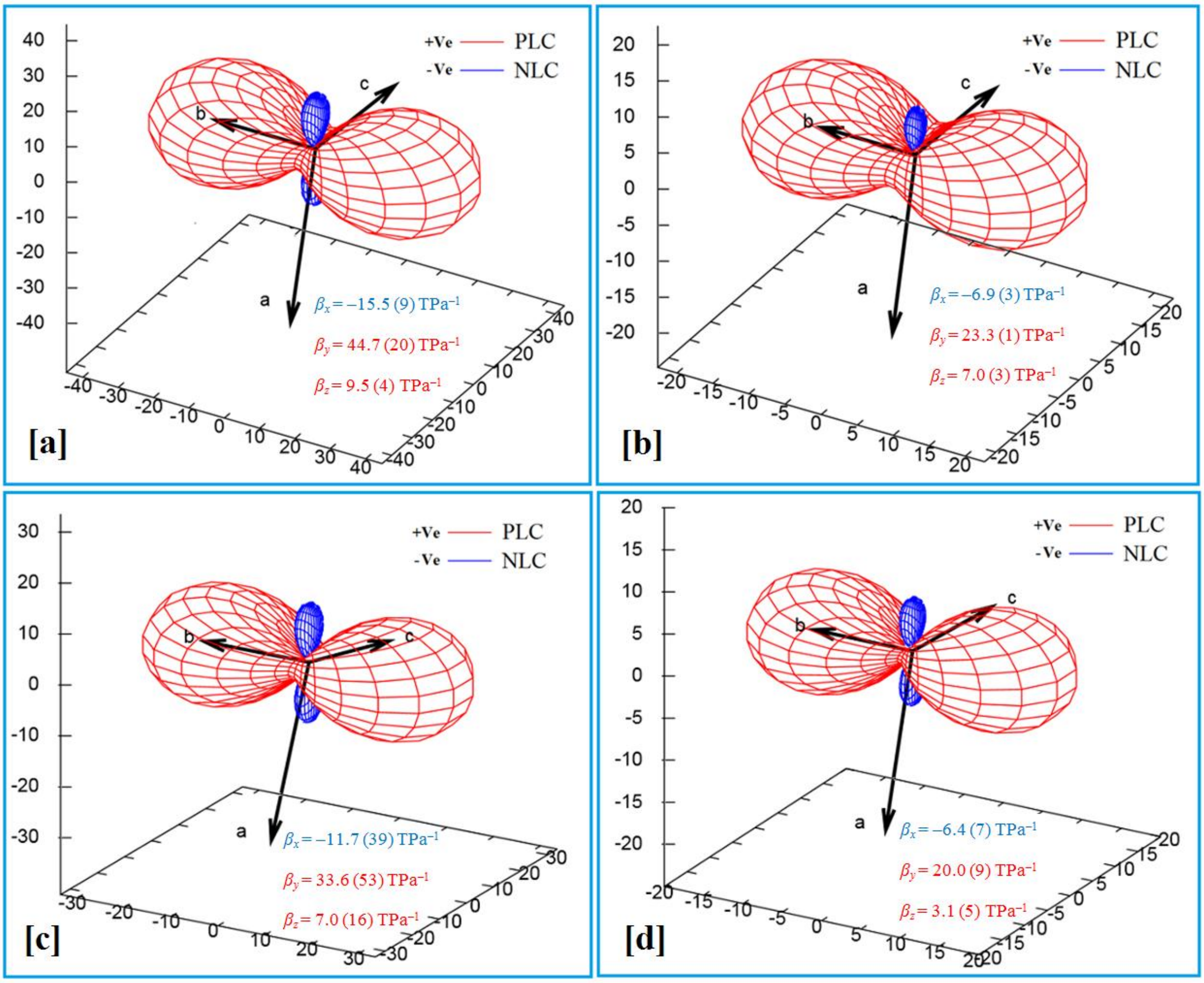

6. **Figure S3.** The average relative bond lengths [a] and bond angles [b] of $[BO_3]^{3-}$/$[BO_4]^{5-}$/$[BiO_4]^{5-}$ groups under different hydrostatic pressures from 0 to 10 GPa.

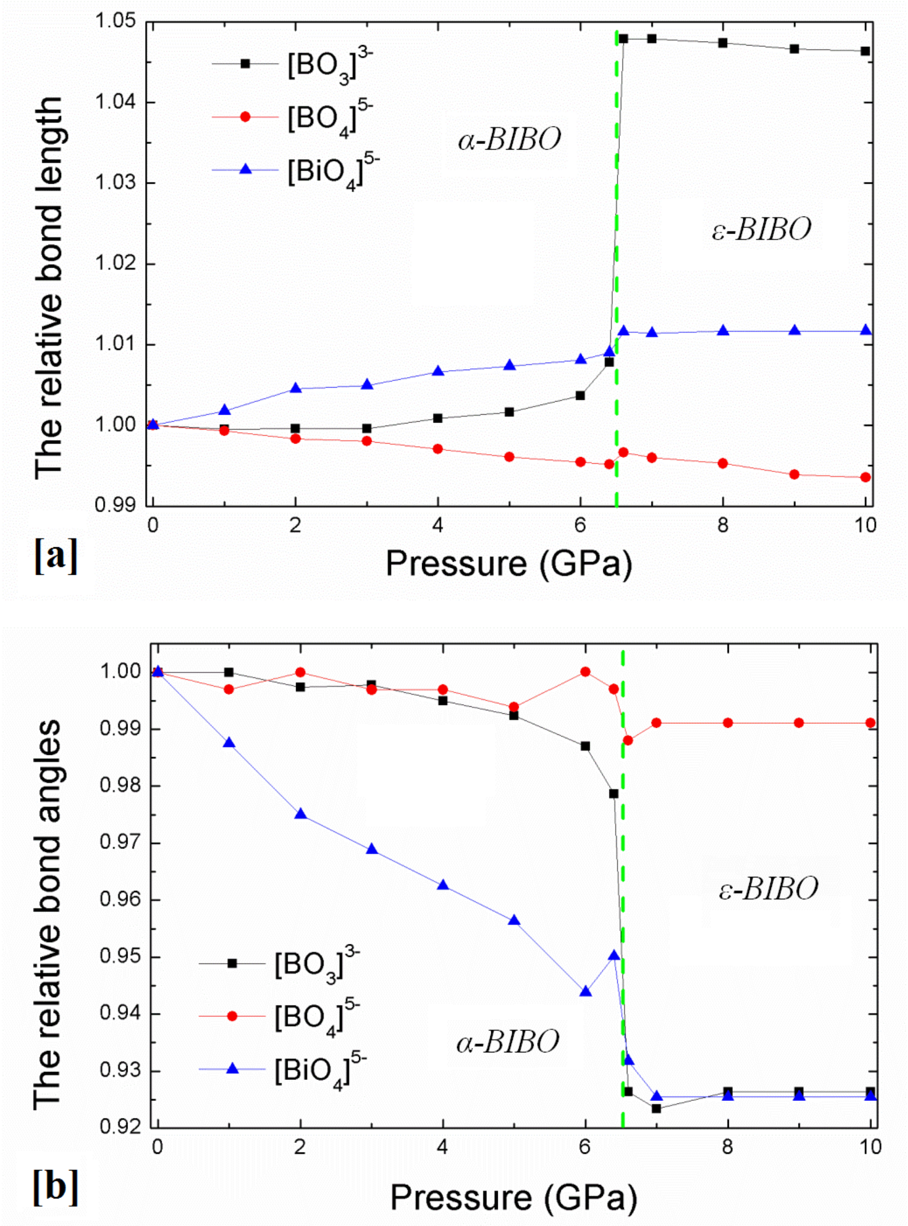

7. **Figure S4.** The atomic structures of BIBO in the *x-y* plane under different pressures: [a] 3.0 GPa, [b] 6.4 GPa, [c] 6.6 GPa, and [d] 9.0 GPa. The electronic lone pairs on the Bi^{3+} atoms of $[BiO_4]^{5-}$ pyramids are illustrated by the blue umbrella-spheres from the electron localization function (ELF) analysis[3]. The angle φ between the $[BO_3]^{3-}$ triangles and $[BO_4]^{5-}$ tetrahedra within the *x-y* plane is indicated.

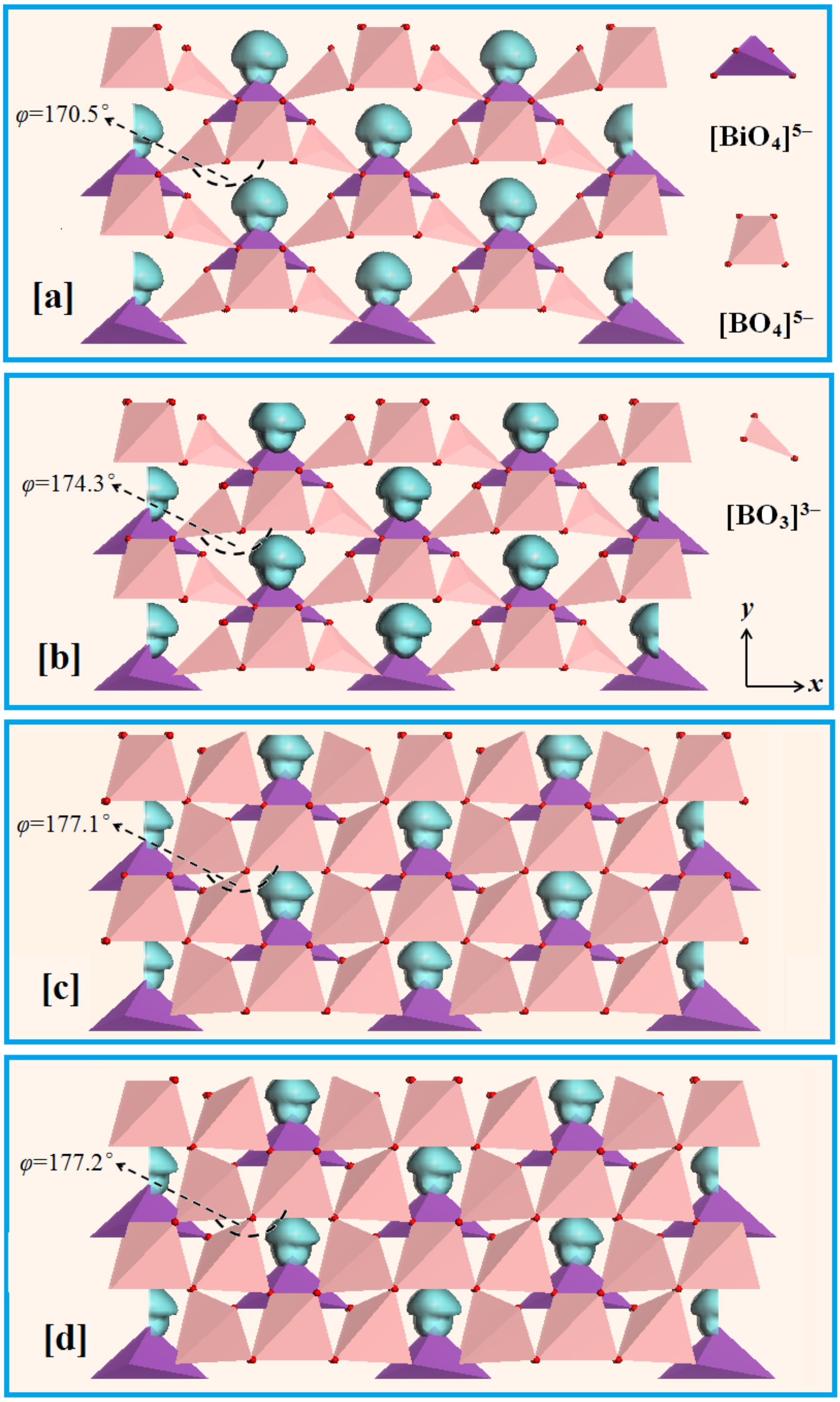

8. Table S4. Calculated elastic constants C_{ij} (GPa) and compressibility coefficients β_x, β_y, β_z, and β_V (GPa^{-1}) of LaB_3O_6.

Calculated elastic constants C_{ij} (GPa); compressibility coefficients β_x, β_y, β_z, and β_V (GPa^{-1})									
C_{11}	282.86	C_{55}	122.15	C_{23}	–3.59	C_{46}	–18.52	β_x	-0.52×10^{-3}
C_{22}	84.81	C_{66}	95.20	C_{15}	–68.68			β_y	12.26×10^{-3}
C_{33}	191.11	C_{12}	62.22	C_{25}	14.39			β_z	5.94×10^{-3}
C_{44}	23.74	C_{13}	75.41	C_{35}	–55.00			β_V	17.68×10^{-3}

9. **Figure S5.** The calculated mechanical properties under pressure in the hypothetical crystal LaB_3O_6 which is geometrically optimized from α-BiB_3O_6 by replacing the Bi^{3+} cations with La^{3+} cations: [a] relative cell constants a/a_0, b/b_0, c/c_0 and V/V_0 (a_0, b_0, c_0 and V_0 are the primitive values at 0 GPa) and [b] compressibility β_x, β_y, β_z and β_V as a function of pressure from 0 to 10 GPa. It is clear that LaB_3O_6 exhibits a much smaller NLC β_x of ~ –2 TPa^{-1} and a much narrower NLC pressure range of ~ 1.5 GPa compared with the β_x value of –27 TPa^{-1} and the pressure range of 6.5 GPa in the "collapsible umbrella" structure in α-BiB_3O_6.

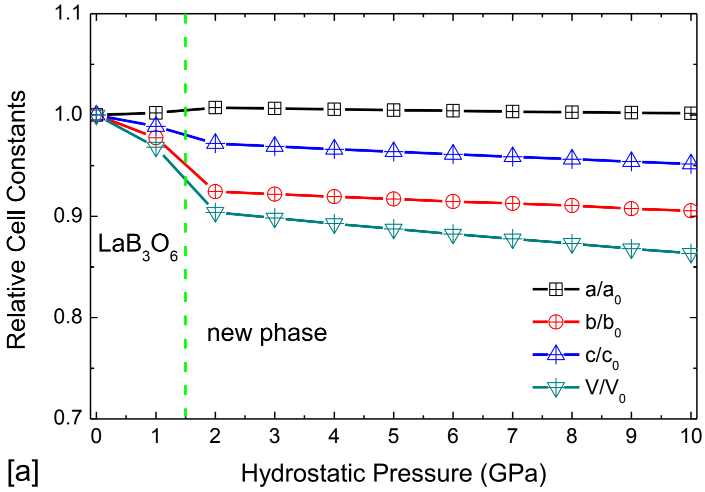

[a]

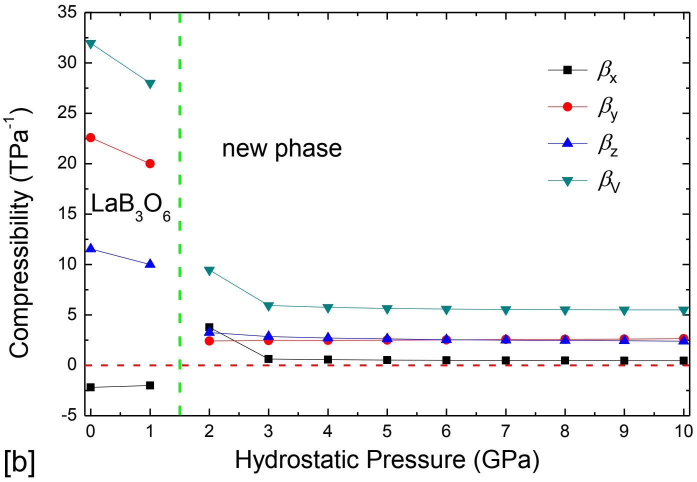

[b]

10. **Figure S6.** The comparison between the experimental XRD pattern and those simulated from the ε-BIBO in Ref.4 [a] and in this work [b]. The red lines represent the experimental XRD patterns, and the black lines represent the simulated data. The difference between experimental and simulated XRD patterns is shown by the grey lines. The R_{wp}, R_p, χ^2 and R_B are 7.10%, 5.33%, 1.09, 3.00% for the former case, and 7.44%, 5.32%, 1.14, 3.33% for the latter case. This indicates that there is no sufficient sensitivity in the data to discriminate the two structures. In fact, since the data resolution is not very good, it is difficult to accurately determine the structures using the Rietveld refinement.

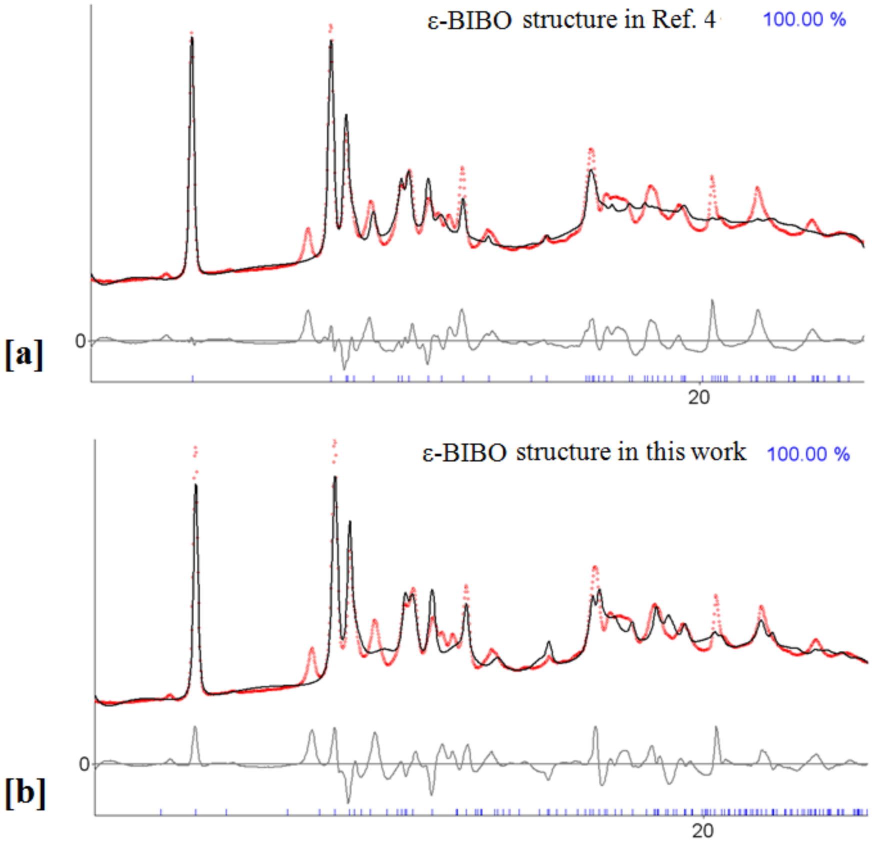

11. References

1. Pfrommer, B. G., Cote, M., Louie, S. G. & Cohen, M. L. Relaxation of crystals with the quasi-Newton method. *J. Comput. Phys.* **131**, 233-240 (1997).

2. Cliffe, M. J. & Goodwin, A. L. PASCal: a principal axis strain calculator for thermal expansion and compressibility determination. *J. Appl. Cryst.* **45**, 1321-1329 (2012).

3. Becke, A. D. & Edgecombe, K. E. A simple measure of electron localization in atomic and molecular-systems. *J. Chem. Phys.* **92**, 5397-5403 (1990).

4. Dinnebier, R. E., Hinrichsen, B., Lennie, A. & Jansen, M. High-pressure crystal structure of the non-linear optical compound BiB_3O_6 from two-dimensional powder diffraction data. *Acta Crystallogr. Sect. B-Struct. Sci.* **65**, 1-10 (2009).
